# The latent difference score model is a viable alternative to arithmetic difference score-based anchor-driven minimal important change calculation

**DOI:** 10.1186/s41687-026-01085-2

**Published:** 2026-05-20

**Authors:** Levent Dumenci, Daniel L. Riddle

**Affiliations:** 1 Temple University, Philadelphia, USA; 2 Virginia Commonwealth University, Richmond, USA

**Keywords:** MIC, Outcome, Prognosis, Pain

## Abstract

**Background:**

Traditional MIC estimates contain measurement error from PROMs. The LDSM offers a viable alternative to estimate MIC controlling for measurement error. We also propose a conceptual framework for considering the multiple steps necessary to calculate anchor-based MICs.

**Methodology:**

Data from KASTPain, a no-effect multi-center randomized clinical trial of 364 participants with knee osteoarthritis and who underwent knee arthroplasty, were used to illustrate the LDSM to estimate MIC. Based on a structural equation modeling framework, we used LDSMs to estimate MICs of three commonly used patient-reported outcomes of pain and functional status.

**Results:**

We reported nine MIC estimates, three PROMs crossed with three post-intervention measurement occasions, from the LDSM. Results from the receiver operating curve analysis indicated that the area under curve was high (median: 0.88 range: 0.77–0.90) for all analyses.

**Conclusions:**

Latent variable modeling of PROM change excludes PROM measurement error and may offer advantages over more traditional forms of MIC estimation.

**Supplementary Information:**

The online version contains supplementary material available at 10.1186/s41687-026-01085-2.

## Introduction

The concept of minimal important change (MIC), sometimes labeled as Minimal Clinically Important Difference (MCID) or Minimal Important Difference (MID) [1], was originally defined by Jaeschke and colleagues [2] as “the smallest difference in score in the domain of interest which patients perceive as beneficial and which would mandate, in the absence of troublesome side effects and excessive cost, a change in the patient’s management.” MIC is considered critically important for interpreting changes in patient reported outcome measures (PROMs) [3–5]. MICs play a critical role in many aspects of medicine. For example, statistical power and sample size determinations heavily rely on MIC estimates in clinical research. In clinical care, MICs inform prognostic judgements regarding recovery and help to guide subsequent courses of action.

A variety of methods exist for calculating MICs including distribution [6], and anchor-based approaches [7] (the focus of the current investigation) but none of these approaches, to our knowledge, account for the measurement error associated with repeated PROM measurement. Despite the importance of MICs in clinical care and research, MIC estimates lack an accounting of random measurement error of the PROM of interest [8]. The primary advantage of using latent difference score models (LDSM) is to remove random measurement error from PROMs in the MIC calculation.

To better understand methods for calculating MICs using latent variable models, we present a conceptual framework for anchor-based approaches to MIC calculation. We see this as a necessary step to specify for the clinician/researcher exactly how MICs are generated so that users will be well-informed to interpret MICs in clinical practice.

### Conceptual framework for determining MIC

**Fig. 1:**
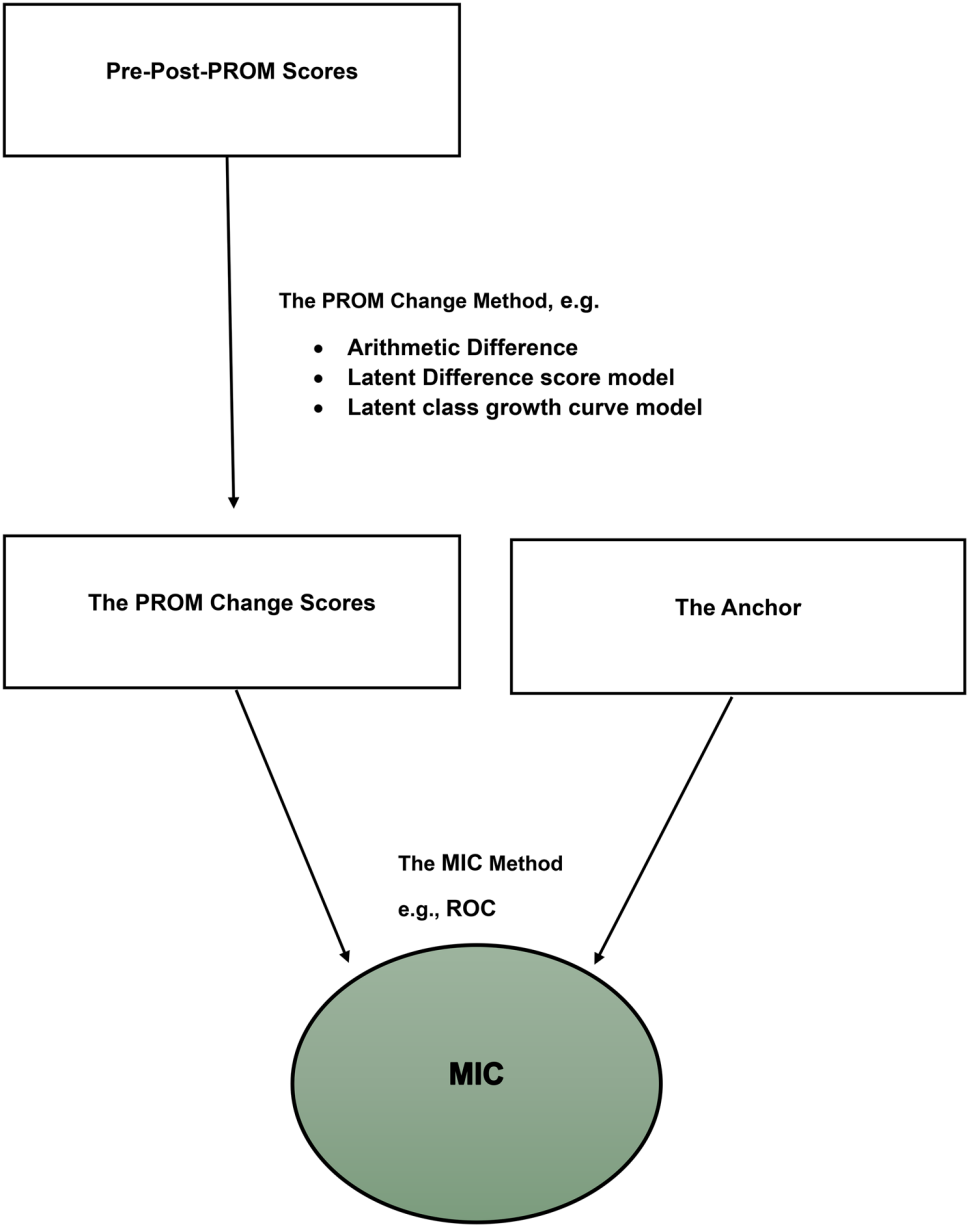
Conceptual model for MIC: The PROM-CAM

The proposed PROM Change-Anchor-Method conceptual framework for MICs (MIC-CAM) focuses on building blocks of anchor-based methods to estimate MICs (Fig. 1). The MIC-CAM framework is designed to aid clinicians, researchers, and policy makers understanding of what a particular MIC represents so that they can make an informed decision about if, or when an MIC can be used. The framework has three components: (a) *The PROM change method*: the method used to obtain PROM change scores from PROMs; (b) *The Anchor*: an anchor question asks about the extent to which the intervention effect is perceived by patients as “beneficial;” and may or may not include information about costs or side-effects, and (c) *The MIC method*: the method uses the PROM change scores and the anchor score to obtain “the smallest difference in score.” The MIC-CAM clearly distinguishes two subtle but important steps: (a) the method used for obtaining the PROM change, most commonly the observed difference between baseline and follow up, and (b) the method utilizing PROM change and the anchor to estimate the MIC.

### PROM change method

PROM change requires a method to estimate the intra-individual change scores from the pre- and post-intervention PROM scores. Thus, these scores contain measurement error, threatening the validity of MIC estimates. We use the term “difference” to refer to the arithmetic difference between the pre- and post-test PROM scores (PROM_*ads*_), both of which contain measurement error. We reserve the term “change” when the PROM change scores do not contain measurement error. Latent variables, by definition, do not contain random measurement error [9], and can be used to estimate true change in the domain of interest.

### The anchor

The anchor aims to tie changes in PROM change scores to change that “patients perceive as beneficial.” An anchor, often framed as a single question with a binary or ordinal response format, has long been considered as a psychometric instrument. However, developers of psychometric instruments are expected to document how the instrument has been developed, along with substantial psychometric evidence for high validity and reliability [10]. We found no studies designed to develop an anchor as a true psychometric instrument. We therefore consider anchors as single-item questions [11] designed to determine if patients perceive the intervention to be worthwhile, not as psychometrically sound instruments designed to confidently measure a construct.

#### The MIC method

The MIC method inputs the PROM change scores and an anchor and outputs the MIC. Receiver operating characteristic (ROC) curves have long been recognized as an acceptable method for cost/benefit analysis in diagnostic decision making and are commonly used to determine MIC [12]. ROC defines MIC as the optimal cut-score that differentiates those with important improvement versus those without important improvement. Optimality is achieved by maximizing the sum of sensitivity and specificity minus one [13]. Alternatives have been proposed [14, 15], but their superiority over ROC has not been demonstrated [7].

Our overriding purpose is to apply patient data from a no-effect randomized clinical trial of 346 patients with knee arthroplasty to illustrate the application of LDSM to estimate MICs (MIC_*lds*_). The LDSMs overcome the PROM random measurement error limitation found with traditional methods of calculating anchor-based MICs. We do not provide a head to head comparison of LDSM to more traditional methods given that there is no gold standard for important change calculation. Without a gold standard, one would not know which method is optimal. However, because LDSM overcomes random measurement error concerns with the outcome of interest, it is our opinion that LDSM will likely provide less biased and more precise MIC estimates with smaller 95% confidence intervals as compared to more traditional arithmetic difference score methods of MIC estimation.

## Methods

### The data source for latent difference score modeling for MIC (MIC_*lds*_)

We utilized data from the KASTPain study, an NIH funded (UM1AR062800) and IRB-approved (IRB HM14326, Virginia Commonwealth University) no-effect knee arthroplasty randomized clinical trial [16], to demonstrate calculation of MIC_*lds*_. Briefly, the following inclusion criteria were used for the current study: patients had (a) a primary knee arthroplasty; (b) a valid response to the anchor question at one or more of the follow-up visits; (c) non-missing 12-month WOMAC Pain, WOMAC Disability [17] or 4-item pain severity [18] ratings scores. The protocol provides a complete description of study [19] while the final trial results reports findings, sample characteristics and inclusion and exclusion criteria [16].

The WOMAC Pain, WOMAC Disability and the 4-item verbal pain rating scales and the anchor was measured at 2-, 6-, and 12-months following knee replacement. The WOMAC Pain scale has strong validity and 5 items with total scores ranging from 0 (no pain with activity) to 20 (extreme pain with activity) while the WOMAC disability scale has 17 items with scores ranging from 0 (no difficulty with activity) to 68 (extreme difficulty with activity) [17]. The 4 item pain scale also has strong psychometric properties [18], and ranges from 0 (no pain) to 10 (pain as intense as you can imagine) summed across 4 items. The anchor [20] was worded in the following way:

*The following question is designed to determine how much your knee problem has changed overall since just prior to your surgery. Compared to just prior to surgery*,* how would you describe your knee these days?*

Patient responses were recorded on a -5 to 5 scale with − 5, 0, and 5 demarcated as vastly worse, no change, and completely recovered, respectively. Scores from − 5 to 0 were coded as 0 (below the threshold) and from 1 to 5 as 1 (at or above the threshold). We believe it is appropriate to combine ratings 1 through 5 because all reflect a patient’s perspective of improvement. In this analysis, the anchor deviates from the Jaeschke et al. definition of MIC as it makes no references to “the absence of troublesome side effects and excessive cost.”

To conduct the modeling, we treated 5- and 10-ordinal category outcomes as continuous and used maximum likelihood method to estimate the LDSMs. Our choice of estimator was based on recommendations from Rhemtulla et al. [21] within the latent variable framework. We used the full information maximum likelihood method to handle missing data.

**Fig. 2:**
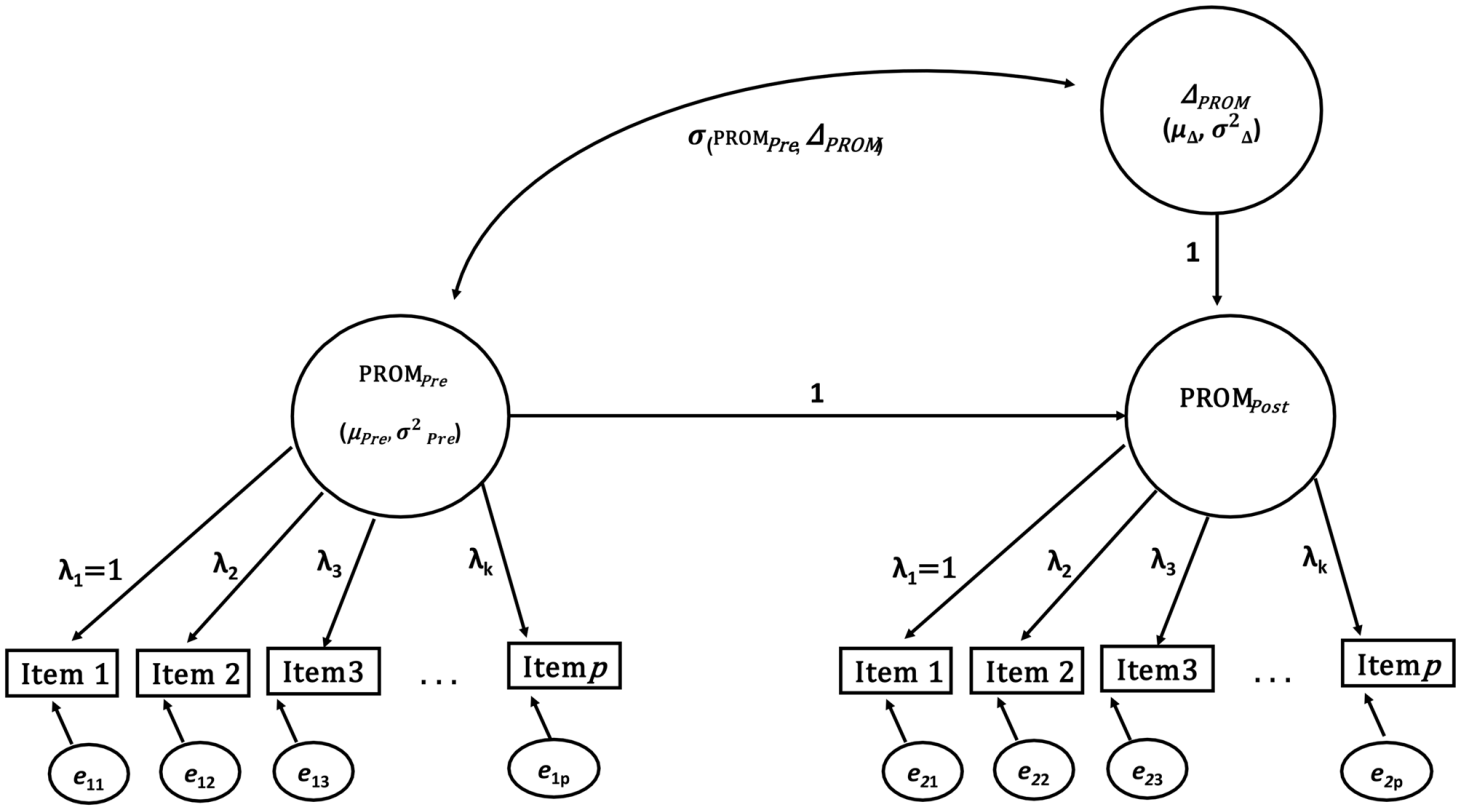
Latent difference score model. Note. PROM_*pre*_ = Pre-intervention latent PROM variable; PROM_*post*_ = Post-intervention latent PROM variable; $\:{\Delta\:}prom$ = latent change variable; µ_*pre*_ – mean of PROM_*pre*_; $\:{\boldsymbol{\sigma\:}}^{2}pre$ = variance of PROM_*pre*_; µ_Δ_ = mean of $\:{\Delta\:}prom$; $\:{\boldsymbol{\sigma\:}}^{2}\boldsymbol{\varDelta\:}$ = variance of $\:{\Delta\:}prom$; λ_j_ = i^*th*^ factor loading; *e*_*ij*_ = residual variance of *j*^*t*h^ item measured at *i*^th^ occasion; $\:\sigma\:$_*(*_*PROM*_*Pre*,_*Δ*_*PROM)*_) = covariance between pre-intervention latent PROM variable and latent change variable

The LDSM, graphically represented in Fig. 2, is one method used to estimate the “difference in score in the domain of interest” corresponding to PROM change in the MIC-CAM. The latent difference is an alternative to the arithmetic difference method. The model implies longitudinal metric invariance, (i.e., equality of factor loadings between the same item measured at baseline and follow up) a testable model attribute assuring that pre and post scores are comparable. Measurement invariance is an untestable assumption of the arithmetic difference method.

The model involves representing the pre and post PROM scores as latent variables (PROM_*Pre*_ and PROM_*Post*_). Item responses are composed into two factors: common across items (or common factor) and specific item factor (i.e., item residual) containing measurement error (e_*ij*_). As specific factors contain measurement error, common factors represent the true levels of the construct (e.g., pain or functional status). To identify the model, either the factor loading of one item or the factor variance is fixed to unity, which effectively sets the scale of the latent variable to the item scale. We set the first item loading to unity for identification. Over a dozen estimators are available to fit this model [22]. To estimate the model, one chooses an estimator appropriate for item distributions. Examples include binary, ordered categorical (ordinal), unordered categorical (nominal), continuous, censored, and zero-inflated item distributions.

In addition to PROM_*Pre*_ and PROM_*Post*_, the PROM change is also conceptualized as a latent variable (Δ_*PROM*_). Variability of each item defining the PROM_*Post*_ is comprised of three parts: true level at baseline (PROM_*Pre*_), true change (Δ_*PROM*_), and item residual (e_*ij*_). Equivalently, the MIC_*lds*_ defines the latent change as PROM_*Post*_ after controlling for true baseline score and measurement error. Whereas the measurement error is orthogonal to both true baseline and true change scores, the covariance between the true baseline and true change, $\:\sigma\:$_(_PROM_*Pre*,_*Δ*_*PROM*)_, is a part of a model parameter.

The MIC_*lds*_ defines the true change as a normally distributed latent variable: Δ_*PROM~*_*N*(µ_Δ*PROM*_, σ^2^_Δ*PROM*_). Because ΔPROM is defined on the same scale as the PROM item through a unit factor loading, it represents latent change in the item’s scale. To express MIC_lds estimates on the observed PROM score metric, ΔPROM was therefore rescaled. This was accomplished by transforming the mean and variance of the ΔPROM factor score estimates to correspond to the expected means and variances of the true change scores implied by the PROM items. It is important to note that MIC itself does not require rescaling of change scores. This rescaling simply allows MIC_lds values to be reported on the original PROM score scale, consistent with traditional MIC reporting. Details of the transformation and MPlus codes for fitting the LDSM are provided in the Supplementary File. The order of operation follows fitting the LSDM, estimating the factor scores for the latent change variable, and then rescaling the factor scores.

The MIC_*lds*_ was used to estimate three MICs for each outcome in outcome units at 2-, 6- and 12-month post-surgery. We used the ROC method to estimate MICs using the anchor question and the rescaled individual latent change scores. The area under curve (AUC) is interpreted in the following way: 0.5 = no discrimination; 0.5–0.7 = poor discrimination; 0.7–0.8: acceptable discrimination; 0.8–0.9 = excellent discrimination; and > 0.9 = outstanding discrimination [23]. All analyses were conducted using Mplus.

## Results

Of 384 participants that underwent surgery in the KASTPain trial, 346 study participants provided 12-month follow-up data and were included in the current study. Mean age of the 346 participants was 63.3 years and 66.8% were female. See Table 1 for a complete sample description. Anchor score distributions for each outcome measure appear in the supplemental file.

**Table 1:** Sample characteristics for KASTPain (*n* = 346)

Variable	KASTPain (*n* = 346)	Missing
Age in yrs, mean (sd)	63.3 (8.1)	0
Female sex, n (%)	231 (66.8)	0
Race/Ethnicity, n (%)	346	0
American Indian or Alaska Native	1 (0.3)	
Asian	8 (2.3)	
Black or African American	120 (34.7)	
Hispanic or Latino/a	13 (3.8)	
White	216 (62.4)	
Other/not reported	2 (0.6)	
Body Mass Index, mean (sd)	32.4 (6.1)	7
Preop WOMAC Pain, mean (sd)	11.3 (3.3)	0
Preop WOMAC Disability, mean (sd)	36.6 (11.4)	0
Comorbidity, mean (sd)	8.5 (4.0)	0
Pre-operative data timing in days		
Preoperative visit, mean (sd)	0	0
First postop visit, mean (sd)	78.8 (23.4)	16
Second postop visit, mean (sd)	200.2 (24.6)	25
Third postop visit, mean (sd)	385.2 (29.6)	0
Score at each visit, mean (sd)		
WOMAC Pain first postop visit	6.1 (3.8)	16
WOMAC Pain second postop visit	4.1 (3.7)	25
WOMAC Pain third postop visit	3.1 (3.7)	0
WOMAC Disability first postop visit	20.0 (12.6)	16
WOMAC Disability sec postop visit	14.3 (12.1)	25
WOMAC Disability third postop visit	11.0 (12.6)	0

### Results for MIC_lds_

Table 2 lists MIC_*lds*_ estimates. The MIC_*lds*_ for WOMAC Pain measured at 2-month post-surgery, for example, is 5. When reporting the MIC_*lds*_ = 5, the PROM, the method used to estimate PROM change, the anchor, and the method (ROC) are accompanied by the explicit definition underlying the MIC, the intervention, and assessment period.

**Table 2:** MIC_*lds*_ estimates for WOMAC Pain, WOMAC Disability, and 4–Item Pain scales

Method	Scale	Months Post Intervention	Score Range	MIC	Latent Difference Score Range		AUC	95% CI	Anchor Performance
					Anchor = 0^#^	Anchor = 1^*^			
LDS	WOMAC Pain	2	0–20	5.00	3.91–5.90	2.59–8.01	0.77	0.70–0.84	Acceptable
		6		6.89	5.27–7.65	5.04–9.25	0.88	0.82–0.93	Excellent
		12		6.98	5.04–9.25	4.50–9.43	0.88	0.80–0.97	Excellent
	WOMAC Dis	2	0–68	15.80	13.49–18.14	14.55–16.84	0.79	0.71–0.88	Acceptable
		6		15.91	12.30–16.44	12.72–19.52	0.85	0.79–0.91	Excellent
		12		14.91	11.71–17.42	1.79–20.46	0.90	0.82–0.99	Outstanding
	4–Item Pain	2	0–40	17.62	11.27–18.15	11.74–25.15	0.85	0.80–0.89	Excellent
		6		25.60	18.31–26.02	18.31–33.86	0.88	0.84–0.93	Excellent
		12		24.81	21.39–30.43	22.75–35.62	0.87	0.78–0.97	Excellent

^#^ An anchor = 0 refers to the range of anchor scores from − 5 (vastly worse) to 0 (no change)

^*^ An anchor = 1 refers to the range of anchor scores from 1 (slightly improved) to 5 (completely recovered)

Note. LDS = latent difference score model

The AUC values and 95% CIs for all nine MIC_*lds*_ estimates are listed in Table 2. The performance of the anchor ranges from acceptable to excellent (AUC range: 0.77–0.90). Anchor performance somewhat improves as the post-assessment period increases.

## Discussion

The MIC_*ads*_ method (i.e., arithmetic difference) has dominated MIC research. In this study, we introduced MIC_*lds*_ as an alternative to the MIC_*ads*_ and provided a demonstration with patient data. In the calculations of MIC_*lds*_, the random measurement error in PROM change estimates were controlled for, a source of criticism historically directed to MIC using arithmetic difference scores [24]. Our conceptual framework explicitly differentiates three steps in the methods used to obtain an MIC. Our analyses show that LDSMs perform well, given the high AUC estimates (median = 0.88; range: 0.77–0.90).

The primary distinction between the arithmetic difference and latent difference methods is the handling of measurement error in PROM scores. The arithmetic difference score ignores measurement error and further compounds measurement error in PROM change scores. Regression to the mean, a common source of error in observational studies, is a consequence of the difference score [25]. Also, the arithmetic difference method does not account for the relationship between PROM_*pre*_ and difference scores. MIC_*lds*_ partials out item-level measurement error from both PROM_*pre*_ and PROM_*post*_ in estimating PROM change while the covariance between the true baseline and true change, $\:\sigma\:$_(_PROM_*Pre*,_*Δ*_*PROM*)_, is estimated as a part of the model parameter overcoming the floor/ceiling effects, baseline severity issues and regression to the mean criticisms of MIC_*ads*_ [26–28].

For *MIC*_*lds*_ = 5 from Table 2, it may seem counterintuitive, for example, that one patient improved 5.90 points (< *MIC*_*lds*_) on WOMAC Pain and rated the anchor as 0 (≤ No improvement) and another patient improved only 2.59 points (> *MIC*_*lds*_) and rated the anchor as 1 (important improvement). However, the former patient’s anchor rating may indicate the unmet expectation of returning to strenuous hiking after surgery, whereas the latter patient met her/his expectation of walking in the home. This counterintuitive finding has implications for the use of *MIC*_*lds*_ in daily practice.

Using WOMAC Pain, for example, arthroplasty practices provide evidence that their patients’ pain reduction, is ≥ 5 over a 2-month period, and aligns with an important improvement at the group level. *MIC*_*lds*_ = 5 also provides an evidence-based benchmark for researchers to conduct sample size determination and statistical power calculations. Clinicians’ interpretation of *MIC*_*lds*_ = 5 for an individual patient is unfounded because each patient has his/her own threshold, expressed in response to an anchor question, and likely differs from 5 depending on idiosyncratic expectations of acceptable outcome. No single number on a psychometric scale, no matter how precisely it was estimated, will likely inform the clinician as to whether meaningful change occurred or whether the clinical course of action needs to be modified for the individual patient. MICs, in our opinion, should not be viewed as a viable substitute for a clinical encounter that discusses whether the patient with his/her unique reasons for undergoing the treatment of interest has benefitted from the intervention. We contend that the PROM change and a patient’s response to an anchor provide valuable tools in clinical encounters to interpret important changes in outcome at the group level but not at the individual patient level.

Regarding our finding of slight increases in MICs over the 12-month period (5 WOMAC Pain points 2 months post-surgery to 6.9 points 6 and 12-months post-surgery), only one other study was found that determined if MICs change over time in patients with knee arthroplasty [29]. Harris and colleagues reported 3-, 12- and 24-month MICs for Oxford knee scores using an arithmetic difference method [29]. They reported MICs of 0.1 at 3 months, 4.2 points at 12 months and 5.1 points at 24 months using the 12-item, 0 (worst score) to 48 (best score) Oxford knee scale [30]. Our prior work [31] suggests that patient recovery trajectories for WOMAC and Oxford scores are similar and, in our view, allow for a limited direct comparison between our KASTPain study and the study of Harris et al. Despite a longer postoperative period of 3 months compared to our study of 2 months, Harris and colleagues reported an MIC of essentially 0 at the 3-month postoperative time point compared to our MIC of 5, obtained 2 months post-surgery [29]. Despite different PROMs, anchors, and time points we find this difference concerning and suggests a potential limitation of the method for calculating MICs in the paper by Harris and colleagues. To support our argument, an approximate 50% average improvement in self-reported pain and function is commonly found 2–3 months post-surgery relative to preoperative values [32], and we suggest patients, on average, expect meaningful improvements in pain and function at these time points. Our study is consistent with these data.

Parametric representation of the change in PROMs as a latent variable is not new within the context of longitudinal IRT [33], as well as within the structural equation modeling traditions [34–35]. The novelty of our study is that we used LDS modeling to accommodate the test-retest design adopted by MIC/MCID researchers and we provided a description of a way to convert the LDS estimates to the PROM units of interest. Latent difference score modeling of change appears to have a clear strength over difference approaches. It defines change as PROM_*post*_ after partialling out true PROM_*pre*_ (i.e., pre-PROM scores without measurement error) and measurement error in PROM_*post*_. Removing measurement error from the true change in PROMs appears to enhance the performance of ROC analysis to obtain MIC using the anchor. The arithmetic difference scoring method is easy to implement, relative to latent variable methods, but this limitation should not, in our opinion, be used to scientifically justify its use.

The MIC-CAM conceptual model provides guidance on further improving MIC calculations in future research. The LDSM provides psychometric advantage to the arithmetic difference method to estimate meaningful change in clinical outcomes. The LDSM overcomes assumptions associated with the arithmetic difference scoring method: The pre and post PROMs are free of random measurement error, and they have invariant measurement properties. Proposals for additional latent variable models will likely follow in future studies. Under the MIC-CAM framework, this study specifically addresses the PROM-change method. We relied on the Jaeschke et al. definition of MCID [2] for anchor recoding. Other anchor definitions may yield different MICD estimates. Also, the issue of random error in single-item anchor questions needs to be effectively dealt with in methodological studies. As for the MIC method, Terluin and colleagues [36] introduced the MIC_pred_ as an extension of the AUC analysis and claimed to be equal to the mean of latent individual MICs [37] in simulation studies. Since its introduction, they further refined the method in subsequent studies [38–39]. The field is open to further improvements to MIC/MCID methods including the removal of PROM pre, post, and change score measurement error via the LDS modeling approach described in the current study.

In conclusion, we provided a theoretical framework and proposed a latent difference score modeling approach to estimate MICs that potentially have psychometric advantages over more traditional MIC methods. We argued that MIC estimates serve two primary functions: to provide evidence-based thresholds for estimating required sample size and power calculations in randomized trials, and to provide group level prognostic benchmarks for at least minimally important benefit. Due to idiosyncratic characteristics of patient care seeking, we also argued that MICs be used to inform patients regarding likely prognosis at the group level but that clinicians refrain from using MICs to make specific judgements regarding individual patient improvement and instead rely on discussions with a patient and her/his unique circumstances to inform the meaning of changes in PROMs.

## Electronic Supplementary Material

Below is the link to the electronic supplementary material.

Supplementary Material 1

## Data Availability

A de-identified dataset will be made available to investigators submitting a complete study proposal made available to authors.
