# Supplementary material for: The latent difference score model is a viable alternative to arithmetic difference score-based anchor-driven minimal important change calculation
Source: J Patient Rep Outcomes. 2026 May 20;10:118. doi: 10.1186/s41687-026-01085-2 (PMC13369083; doi:10.1186/s41687-026-01085-2)
Supplement: Supplementary file 1 — Supplementary Material 1 [file 41687_2026_1085_MOESM1_ESM.docx]

**Supplementary File.**

**Page 1. Rescaling true change scores to estimate MCID*_lds_***

**Page 2. Anchor score distributions for each outcome measure**

**Page 3. MPlus input file to estimate MCID*_lds_***

**Page 4. IBM SPSS syntax file to rescale true change scores into the PROM scale**

From **Figure 2**, the *j*^th^ item (*j* = 1 to *p*) used to measure PROM*_Post_* is expressed as the sum of true baseline (PROM*_pre_*), true change (Δ*_prom_*), and item residual ( *e_j_*.):

*Y*_2j_ = λ_j_(PROM*_pre_*)+ λ_j_(Δ*_prom_*) + *e_j_*.

The mean of true change over *p* items are given as

$\sum_{j=1}^{p} \varepsilon(\lambda j(\Delta prom)$)

where $\varepsilon$ is the expectation operator.

The rescaled mean of true change **(μ*_rt_*)** becomes

$(\sum_{j=1}^{p} \lambda j)$(μ_Δ_).

The variance of true change over *p* items are expressed as

Var$(\sum_{j=1}^{p} (\lambda j(\Delta prom)))$.

The rescaled variance of true change ($\boldsymbol{\sigma}^{\boldsymbol{2}}$_rt_) becomes

$(\sum_{j=1}^{p} ((\lambda j)(\lambda j)$($\boldsymbol{\sigma}^{\boldsymbol{2}}\boldsymbol{\Delta}$))+(2$\boldsymbol{\sigma}$(***PROM_Pre,_ Δ_PROM_***))).

Using the z-transformed factor score estimates (*z_fs_*), the rescaled true change score for each person **(Y*_rt_*)** becomes

μ*_rt_* + ((*z_fs_*)(√$\boldsymbol{\sigma}^{\boldsymbol{2}}$_rt_)).

Table 1. Anchor scale categories of WOMAC Pain, WOMAC Disability and 4-item pain scores collected at 2-, 6, and 12-months.

| Scale and MCID score | 2-month outcome | | 6-month outcome | | 12-month outcome | |
| --- | --- | --- | --- | --- | --- | --- |
|  | Mean change | n | Mean change | n | Mean change | n |
| **WOMAC Pain** |  |  |  |  |  |  |
| +5 (completely recovered) | 8.02 | 50 | 8.92 | 100 | 9.69 | 156 |
| +4 | 6.00 | 88 | 7.91 | 110 | 8.16 | 100 |
| +3 | 4.87 | 127 | 6.49 | 69 | 7.15 | 55 |
| +2 | 3.91 | 42 | 5.10 | 21 | 4.35 | 17 |
| +1 | 3.00 | 8 | 5.00 | 6 | 4.00 | 1 |
| 0 (no change) | 1.33 | 6 | 3.60 | 10 | 1.75 | 4 |
| -1 | 0.80 | 5 | 2.00 | 1 | 9.00 | 1 |
| -2 | 0.75 | 4 | 2.50 | 2 | 2.00 | 1 |
| -3 | 4.80 | 5 | 3.00 | 5 | 6.00 | 3 |
| -4 | 2.33 | 3 | 2.00 | 2 | 0.00 | 1 |
| -5 (vastly worse) | 2.25 | 8 | 1.29 | 7 | 0.57 | 7 |
|  |  |  |  |  |  |  |
| **WOMAC Disability** |  |  |  |  |  |  |
| +5 (completely recovered) | 24.04 | 50 | 28.00 | 100 | 31.15 | 156 |
| +4 | 19.68 | 88 | 23.82 | 110 | 26.14 | 100 |
| +3 | 15.70 | 127 | 20.55 | 69 | 19.22 | 55 |
| +2 | 12.21 | 42 | 16.33 | 21 | 12.06 | 17 |
| +1 | 18.00 | 8 | 12.33 | 6 | 23.00 | 1 |
| 0 (no change) | 5.17 | 6 | 7.40 | 10 | -1.00 | 4 |
| -1 | 1.60 | 5 | 7.00 | 1 | 38.00 | 1 |
| -2 | 4.50 | 4 | 15.50 | 2 | 6.00 | 1 |
| -3 | 9.40 | 5 | 7.80 | 5 | 11.67 | 3 |
| -4 | 8.33 | 3 | -4.00 | 2 | -2.00 | 1 |
| -5 (vastly worse) | 4.38 | 8 | 7.29 | 7 | -0.29 | 7 |
|  |  |  |  |  |  |  |
| **4-item Pain** |  |  |  |  |  |  |
| +5 (completely recovered) | 4.39 | 50 | 4.69 | 100 | 5.15 | 156 |
| +4 | 3.33 | 88 | 4.46 | 110 | 4.33 | 100 |
| +3 | 2.54 | 127 | 3.03 | 69 | 2.95 | 55 |
| +2 | 2.57 | 42 | 2.65 | 21 | 2.79 | 17 |
| +1 | 2.34 | 8 | 3.08 | 6 | 1.25 | 1 |
| 0 (no change) | 0.67 | 6 | 1.55 | 10 | 0.13 | 4 |
| -1 | 0.40 | 5 | 0.50 | 1 | 5.50 | 1 |
| -2 | 0.69 | 4 | 0.88 | 2 | 2.75 | 1 |
| -3 | 1.20 | 5 | 1.45 | 5 | 1.58 | 3 |
| -4 | 0.58 | 3 | -0.63 | 2 | 0.50 | 1 |
| -5 (vastly worse) | 0.66 | 8 | 0.35 | 7 | 0.14 | 7 |

TITLE: Estimating latent difference scores for WOMAC Pain pre - 2-month post

DATA: FILE = wpb2m.dat;

FORMAT = A7,88F1,16F2,3F1;

VARIABLE:

NAMES ARE sid

WP0001 WP0002 WP0003 WP0004 WP0005 WP0201 WP0202 WP0203 WP0204 WP0205

WP0601 WP0602 WP0603 WP0604 WP0605 WP1201 WP1202 WP1203 WP1204 WP1205

WD0001 WD0002 WD0003 WD0004 WD0005 WD0006 WD0007 WD0008 WD0009 WD0010

WD0011 WD0012 WD0013 WD0014 WD0015 WD0016 WD0017

WD0201 WD0202 WD0203 WD0204 WD0206 WD0207 WD0208 WD0209 WD0210

WD0211 WD0212 WD0213 WD0214 WD0215 WD0216 WD0217

WD0601 WD0602 WD0603 WD0604 WD0605 WD0606 WD0607 WD0608 WD0609 WD0610

WD0611 WD0612 WD0613 WD0614 WD0615 WD0616 WD0617

WD1201 WD1202 WD1203 WD1204 WD1205 WD1206 WD1207 WD1208 WD1209 WD1210

WD1211 WD1212 WD1213 WD1214 WD1215 WD1216 WD1217

CP0001 CP0002 CP0003 CP0004 CP0201 CP0202 CP0203 CP0204

CP0601 CP0602 CP0603 CP0604 CP1201 CP1202 CP1203 CP1204

GRC2 GRC6 GRC12;

USEVARIABLES ARE

WP0001 WP0002 WP0003 WP0004 WP0005

WP0201 WP0202 WP0203 WP0204 WP0205;

MISSING ARE BLANK;

ANALYSIS: ESTIMATOR = ML;

MODEL:

F1 by Wp0001@1

WP0002-WP0005 (1-4); !Equality of factor loadings

F2 by Wp0201@1

WP0202-WP0205 (1-4);

[WP0001-WP0005@0 WP0201-WP0205@0]; !Equality of factor loadings

WP0001-WP0005 (5-9);

WP0201-WP0205 (5-9);

F2 on F1@1;

LC by F2@1; !Latent difference variable

F2@0;

[F2@0 F1 LC];

OUTPUT: STANDARDIZED;

SAVEDATA: file=wpb2mfs.dat; save=fscores; !Saving latent difference score estimates

!IBM SPSS input file for converting the latent difference score estimates into the original PROM metric

compute sumFLwp2=5.185. !Sum of factor loadings

compute sumsqFLwp2=2.674635. !Sum of squared factor loadings

compute muFSwp2=-1.054127167630056. !mean of latent difference factor scores

compute sdFSwp2=0.557139272793407. !sd of latent difference factor scores

compute muDeltawp2=-1.054. !mean of latent difference variable

compute varDeltawp2=0.427. !variance of latent difference variable

compute zwp2=(fswp2-mufswp2)/sdfswp2. !transforming latent difference factor scores with mean=0 and sd=1

compute mtwp2=sumFLwp2*muDeltawp2. !Setting the mean of transformed latent difference factor scores

compute sdtwp2=sqrt(sumsqFLwp2*varDeltawp2). !Setting the sd of transformed latent difference factor scores

compute scldwp2=mtwp2+(zwp2*sdtwp2). !Rescaled latent difference factor scores

execute.

STROBE Statement—Checklist of items that should be included in reports of ***cohort studies***

|  | Item No | Recommendation | Page No |
| --- | --- | --- | --- |
| **Title and abstract** | 1 | (*a*) Indicate the study’s design with a commonly used term in the title or the abstract | 1 |
|  |  | (*b*) Provide in the abstract an informative and balanced summary of what was done and what was found | 3 |
| Introduction | | | |
| Background/rationale | 2 | Explain the scientific background and rationale for the investigation being reported | 4 |
| Objectives | 3 | State specific objectives, including any prespecified hypotheses | 4 |
| Methods | | | |
| Study design | 4 | Present key elements of study design early in the paper | 5-8 |
| Setting | 5 | Describe the setting, locations, and relevant dates, including periods of recruitment, exposure, follow-up, and data collection | 8 |
| Participants | 6 | (*a*) Give the eligibility criteria, and the sources and methods of selection of participants. Describe methods of follow-up | 8 |
|  |  | (*b*) For matched studies, give matching criteria and number of exposed and unexposed |  |
| Variables | 7 | Clearly define all outcomes, exposures, predictors, potential confounders, and effect modifiers. Give diagnostic criteria, if applicable | 8-9 |
| Data sources/ measurement | 8* | For each variable of interest, give sources of data and details of methods of assessment (measurement). Describe comparability of assessment methods if there is more than one group | 8-9 |
| Bias | 9 | Describe any efforts to address potential sources of bias | na |
| Study size | 10 | Explain how the study size was arrived at | 8 |
| Quantitative variables | 11 | Explain how quantitative variables were handled in the analyses. If applicable, describe which groupings were chosen and why | 7,9 |
| Statistical methods | 12 | (*a*) Describe all statistical methods, including those used to control for confounding |  |
|  |  | (*b*) Describe any methods used to examine subgroups and interactions |  |
|  |  | (*c*) Explain how missing data were addressed |  |
|  |  | (*d*) If applicable, explain how loss to follow-up was addressed | 5-9 |
|  |  | (*e*) Describe any sensitivity analyses |  |
| Results | | |  |
| Participants | 13* | (a) Report numbers of individuals at each stage of study—eg numbers potentially eligible, examined for eligibility, confirmed eligible, included in the study, completing follow-up, and analysed | 10 |
|  |  | (b) Give reasons for non-participation at each stage |  |
|  |  | (c) Consider use of a flow diagram |  |
| Descriptive data | 14* | (a) Give characteristics of study participants (eg demographic, clinical, social) and information on exposures and potential confounders | Table 1 |
|  |  | (b) Indicate number of participants with missing data for each variable of interest |  |
|  |  | (c) Summarise follow-up time (eg, average and total amount) |  |
| Outcome data | 15* | Report numbers of outcome events or summary measures over time | Table 2, supp file |

| Main results | 16 | (*a*) Give unadjusted estimates and, if applicable, confounder-adjusted estimates and their precision (eg, 95% confidence interval). Make clear which confounders were adjusted for and why they were included | Table 2 |
| --- | --- | --- | --- |
|  |  | (*b*) Report category boundaries when continuous variables were categorized | 9 |
|  |  | (*c*) If relevant, consider translating estimates of relative risk into absolute risk for a meaningful time period |  |
| Other analyses | 17 | Report other analyses done—eg analyses of subgroups and interactions, and sensitivity analyses | na |
| Discussion | | | |
| Key results | 18 | Summarise key results with reference to study objectives | 10-11 |
| Limitations | 19 | Discuss limitations of the study, taking into account sources of potential bias or imprecision. Discuss both direction and magnitude of any potential bias | 13 |
| Interpretation | 20 | Give a cautious overall interpretation of results considering objectives, limitations, multiplicity of analyses, results from similar studies, and other relevant evidence | 13-14 |
| Generalisability | 21 | Discuss the generalisability (external validity) of the study results | na |
| Other information | | | |
| Funding | 22 | Give the source of funding and the role of the funders for the present study and, if applicable, for the original study on which the present article is based | 15 |

*Give information separately for exposed and unexposed groups.

**Note:** An Explanation and Elaboration article discusses each checklist item and gives methodological background and published examples of transparent reporting. The STROBE checklist is best used in conjunction with this article (freely available on the Web sites of PLoS Medicine at http://www.plosmedicine.org/, Annals of Internal Medicine at http://www.annals.org/, and Epidemiology at http://www.epidem.com/). Information on the STROBE Initiative is available at http://www.strobe-statement.org.
